# Fasting Leptin Is a Metabolic Determinant of Food Reward in Overweight and Obese Individuals during Chronic Aerobic Exercise Training

**DOI:** 10.1155/2014/323728

**Published:** 2014-03-11

**Authors:** Mark Hopkins, Catherine Gibbons, Phillipa Caudwell, Dominic-Luc Webb, Per M. Hellström, Erik Näslund, John E. Blundell, Graham Finlayson

**Affiliations:** ^1^Academy of Sport and Physical Activity, Faculty of Health and Wellbeing, Sheffield Hallam University, Sheffield S10 2BP, UK; ^2^Institute of Psychological Sciences, Faculty of Medicine and Health, University of Leeds, Leeds LS2 9JT, UK; ^3^Department of Medical Sciences, Gastroenterology and Hepatology, Uppsala University, Uppsala, Sweden; ^4^Department of Medical Sciences, Uppsala University, Uppsala, Sweden; ^5^Division of Surgery, Department of Clinical Sciences, Danderyd Hospital, Karolinska Institute, Stockholm, Sweden

## Abstract

Changes in food reward have been implicated in exercise-induced compensatory eating behaviour. However, the underlying mechanisms of food reward, and the physiological correlates of exercise-induced changes in food reward, are unknown. *Methods.* Forty-six overweight and obese individuals completed 12 weeks of aerobic exercise. Body composition, food intake, and fasting metabolic-related hormones were measured at baseline, week six, and postintervention. On separate days, the reward value of high-and-low-fat food (explicit liking and implicit wanting) was also assessed at baseline, week six, and postintervention. *Results.* Following the intervention, FM, FFM, and VO_2peak_ improved significantly, while fasting leptin decreased. However, food intake or reward did not change significantly. Cross-sectional analyses indicated that FM (*P* = 0.022) and FFM (*P* = 0.046) were associated with explicit liking for high-fat food, but implicit wanting was associated with FM only (*P* = 0.005). Fasting leptin was associated with liking (*P* = 0.023) and wanting (*P* = 0.021) for high-fat food. Furthermore, a greater exercise-induced decline in fasting leptin was associated with increased liking (*P* = 0.018). *Conclusion.* These data indicate that food reward has a number of physiological correlates. In particular, fasting leptin appears to play an active role in mediating food reward during exercise-induced weight loss.

## 1. Introduction

Day-to-day food intake involves the coordination of both homeostatic and nonhomeostatic signals in the overall expression of eating behaviour [1]. Homeostatic feeding is often described through a series of physiological processes that initiate and terminate feeding (i.e., satiation) and suppress intermeal hunger (i.e., satiety) [2]. This pattern of eating is thought to be driven by tonic and episodic inhibitory signals (arising from adipose tissue and the gastrointestinal tract) that modulate an intrinsic excitatory drive to eat [3]. However, extrinsic determinants of eating behaviour such as food palatability and hedonic reward, linked closely to perceived fat and energy content of food, interact with these homeostatic mechanisms with the potential to enhance or undermine appetite control [4]. Recent attention has started to focus on the hedonic determinants of eating behaviour and has highlighted the importance of distinguishing liking (i.e., the perceived pleasurable sensory properties of food) from wanting (i.e., the attraction towards a specific food over available alternatives) [5, 6]. Both components of food reward are thought to act in parallel to facilitate eating behaviour [7]. Indeed, heightened liking and wanting for food have been noted in overweight and obese individuals [8] and those who demonstrate binge eating [9].

Changes in food reward may also play a role in compensatory eating behaviour following exercise [10–12]. For example, following 50 minutes of cycling, lean women who overconsumed relative to the energy cost of exercise exhibited increased wanting for food compared to those who did not exhibit postexercise compensatory eating [10]. Furthermore, overweight and obese individuals who exhibited an immediate postexercise increase in explicit liking and wanting for food (particularly, high fat sweet foods) demonstrated smaller fat mass losses following a program of aerobic exercise training [11]. However, while these findings suggest that exercise-induced changes in food reward influence compensatory eating behaviour, the physiological processes that influence food reward are not well understood. Given that appetite control is a psychobiological process [13], it is plausible that a prolonged and potent metabolic stimulus such as aerobic exercise training would be reflected in an increased motivational drive for high energy yielding foods. However, whether adaptive changes in metabolism influence food reward during chronic exercise training has yet to be examined. Therefore, this study aimed to examine whether exercise-induced changes in body composition or metabolic-related hormones influenced food reward in overweight and obese individuals during 12 weeks of aerobic exercise.

## 2. Materials and Methods

### 2.1. Participants

Forty-six overweight and obese individuals participated in the present study (30 females, BMI = 30.8 ± 3.5 kg/m^2^; 16 males, BMI = 30.5 ± 4.7 kg/m^2^). Participants were recruited from the University of Leeds, UK, and surrounding areas using poster advertisements and recruitment emails. Participants were initially physically inactive (≤2 hrs.wk^−1^ of exercise over the previous six months), weight stable (±2 kg for the previous three months), nonsmokers, and not taking medication known to effect metabolism or appetite. At baseline mean dietary restraint and disinhibition scores, as measured using the Three-Factor Eating Questionnaire [14], were 7.21 ± 0.55 and 8.10 ± 0.50, respectively, which are within the normal ranges for healthy adults [15]. The study was conducted in accordance with the Declaration of Helsinki (1964), and all participants provided written informed consent before taking part. In addition, ethical approval was granted by the Institute of Psychological Science's Ethics Board, University of Leeds, and the Leeds West NHS Research Ethics Committee (09/H1307/7).

### 2.2. Study Design

Participants completed a 12-week supervised aerobic exercise program designed to expend 2500 kcal.wk^−1^. Body composition, food intake, and fasting metabolic-related hormones (glucose, insulin, and leptin) were measured at baseline, week six, and postintervention. In addition, explicit liking and implicit wanting for a standardised array of high fat and low fat foods were assessed before a fixed-energy meal, at baseline, week six, and postintervention, using a validated computer based task, for example, the Leeds Food Preference Questionnaire [7].

### 2.3. Exercise Protocol

Participants completed a 12-week aerobic exercise program in which they exercised five days per week, expending 500 kcal per session at 70% of age-predicted maximum heart rate. All exercise sessions were supervised in the research laboratory, and participants could choose from a range of exercise modes (running, cycling, rowing, or stepping). Individual exercise prescriptions were calculated using standard stoichiometric equations [16], based on the relationship between heart rate and VO_2_/VCO_2_ during a maximal incremental treadmill test. To account for changes in cardiovascular fitness during the intervention, the incremental test was performed at baseline, week six, and postintervention, with the exercise prescription adjusted accordingly. To verify and record the duration and intensity of exercise, participants wore a heart rate monitor during each session (Polar RS400, Polar, Kempele, Finland). Total exercise-induced energy expenditure during the intervention was 27960 ± 3479 kcal, which represented >98% of the prescribed exercise-induced energy expenditure.

### 2.4. Physiological Measures

At baseline, week six, and postintervention, venous blood, body composition, and maximal aerobic capacity were measured in the morning (7–9am) following an overnight fast (10–12 hrs). Baseline measures were taken prior to the start of the intervention, while postintervention measures were taken upon completion of the exercise intervention (a minimum of 48 hrs after the final exercise bout and within one week of finishing the intervention). Body composition was measured using air-displacement plethysmography (BOD POD Body Composition System, Life Measurement, Inc., Concord, USA). After voiding, participants were weighed (to the nearest 0.01 kg) and instructed to sit in the BOD POD. Measurements were taken according to manufacturers' instructions, with thoracic gas volumes estimated using the manufacturer's software. In addition, the fat mass index (FMI; fat mass/height^2^) and the fat-free mass index (FFMI; fat-free mass/height^2^) were calculated from these body composition data. Maximal aerobic capacity (VO_2peak_) was determined using a validated maximal incremental treadmill test [17], with expired air (Sensormedics Vmax29, Yorba Linda, USA) and heart rate (Polar RS400, Polar, Kempele, Finland) measured continuously. The respiratory and heart rate data from this test were also used to calculate the exercise prescriptions used in the exercise intervention.

### 2.5. Metabolic- and Appetite-Related Hormones

Fasting glucose, insulin, and leptin were measured at baseline, week six, and postintervention in a subsample of 32 participants who completed the exercise intervention. Fasting venous blood samples were collected into EDTA-containing Monovette tubes. After collection, blood samples were centrifuged for 10 min at 4°C at 3500 rpm and were immediately pipetted into Eppendorf tubes and stored at −80°C until analysis. Insulin and leptin were analysed using a magnetic bead based multiples kit (Millipore, Billerica, MA, USA). Furthermore, insulin resistance was calculated using the homeostatic model of assessment (HOMA) [18].

### 2.6. Assessment of Food Reward and Food Intake

A laboratory-based test meal protocol was used to measure food intake at baseline, week six, and postintervention. At each time point, participants consumed test meals at 4-hour intervals. No exercise was performed on these days. A detailed description of the foods provided can be found elsewhere [19]. Meals consisted of an individualised energy breakfast (*ad libitum* at baseline and then fixed at baseline levels for the remainder of intervention), a fixed-energy lunch (800 kcal), and an* ad libitum* dinner meal. After the dinner meal, participants were free to leave the research unit but were given an* ad libitum* snack box of foods to consume if desired during the evening. All meals consumed in the research unit were eaten in isolation, with participants instructed to eat as much or as little as they wanted until comfortably full (during* ad libitum* meal consumption).

Prior to the lunch test meal, food reward was assessed using the Leeds Food Preference Questionnaire (LFPQ; [7]). The LFPQ measured liking and wanting for foods according to differences in fat content (i.e., >50% or <20% energy from fat). Each food category was represented by 8 photographs of ready-to-eat foods that were matched for familiarity, taste, protein, and acceptability. Firstly, to measure “implicit wanting” a forced-choice paradigm was used in which participants were presented with two foods from different categories and were required to press a key as quickly as possible to indicate which food “they most want to eat at that moment.” This was repeated until all food photograph pairs had been presented. Following Dalton et al. [20], the parameters were set as 96 randomised food pair trials presented in three blocks, with each stimulus appearing 8 times. Stimuli were presented until a valid response was detected up to a maximum of 4000 ms with a 500 ms washout between presentations in which a central fixation cross was displayed. Mean response times for choices outside of each food category, adjusted for choice frequency, were subtracted from response times for choices towards each category, adjusted for frequency. Therefore positive scores for a specific category indicated a more rapid preference (i.e., “implicit wanting”). Secondly, to measure explicit liking, the food images were presented individually in randomised order, and the participant rated the extent to which they liked each food image presented to them using a 100 mm visual analogue scale; for example, how pleasant would it be to taste this food now? Mean scores for high fat and low fat food categories were calculated. The LFPQ has been shown to demonstrate reliable immediate postexercise and postmeal changes [21] and is a good predictor of food choice and intake in laboratory and community settings [22, 23].

### 2.7. Statistical Analysis

Data are reported as mean ± SEM throughout. Statistical analyses were performed using IBM SPSS for Windows (Chicago, Illinois, Version 21). For food reward measures, mean scores for high fat and low fat categories were computed for implicit wanting and explicit liking outcomes. Mean low fat scores were then subtracted from the mean for high fat scores to provide a composite score representing reward value for high fat relative to low fat food for both liking and wanting. Using this approach, a positive score indicated greater liking or wanting for high fat foods over low fat foods; a negative score indicated greater liking or wanting for low fat foods over high fat foods; and a score of zero indicated an equal liking or wanting for high and low fat foods. Scores on each food reward outcome were calculated at baseline, week six, and postintervention and analysed using one-way repeated measures ANOVAs.

Changes in body composition, metabolic-related hormones, and total daily energy intake were examined using one-way repeated measures ANOVAs. Where appropriate, Greenhouse-Geisser probability levels were used to adjust for sphericity, and Bonferroni adjustments were applied to control for multiple post hoc comparisons. To test for associations between physiological variables and food reward, Pearson partial correlation coefficients were used, controlling for sex. Firstly, cross-sectional models were examined using mean scores on each variable collapsed across the three time points of the exercise intervention (i.e., baseline, week six, and postintervention). Secondly, associations between changes in physiological variables and changes in explicit liking and implicit wanting following the exercise intervention were performed. Change variables were calculated by subtracting baseline scores from postintervention scores. To control for confounding effects of body composition, metabolic hormones were tested both with and without adjustment for adiposity by dividing by percentage body fat.

## 3. Results

### 3.1. Changes in Body Composition and Metabolism following the Exercise Intervention

As can be seen in Table 1, there was a significant reduction in body mass (−1.72 ± 0.41 kg; *P* < 0.001), fat mass (−2.23 ± 0.38 kg; *P* < 0.001), and percentage body fat (−1.90 ± 0.22%; *P* < 0.001) following the exercise intervention, while fat-free mass was preserved at baseline levels (+0.52 ± 0.17 kg; *P* = 0.081). Furthermore, FMI decreased (−0.76 ± 0.14 kg/m^2^; *P* < 0.001) and FFMI increased significantly (0.17 ± 0.62 kg/m^2^; *P* < 0.01). VO_2peak_ increased from 33.33 ± 1.17 mL.kg.min^−1^ at baseline to 39.16 ± 0.09 mL.kg.min^−1^ after intervention (*P* < 0.001). There were no significant changes in fasting glucose (−0.20 ± 0.25 mmol.L^−1^; *P* = 0.415) or fasting insulin (−42.98 ± 82.94 ng.L^−1^; *P* = 0.230) following the exercise intervention. However, fasting leptin decreased significantly following the exercise intervention (−6215.93 ± 3076.37 ng.L^−1^; *P* = 0.023).

### 3.2. Changes in Food Intake and Food Reward following the Exercise Intervention

Table 2 shows that total daily energy intake, explicit liking, and implicit wanting for high fat food did not differ significantly between baseline and week 6 or baseline and postintervention. There was a nonsignificant trend for implicit wanting to shift from a small bias for high fat food at baseline, towards a bias for low fat food following the intervention (*P* = 0.114).

### 3.3. Physiological Correlates of Food Reward: Cross-Sectional Associations

As can be seen in Table 3, liking for high fat food was positively associated with body mass (*P* = 0.008) and fat mass (*P* = 0.022) and marginally associated with fat-free mass (*P* = 0.046). However, there were no significant associations between these components of body composition when adjusted for height (i.e., FMI or FFMI). Wanting for high fat foods was also positively associated with body mass (*P* = 0.004), fat mass (*P* = 0.005), and FMI (*P* = 0.018), but not fat-free mass (*P* = 0.129) or FFMI (*P* = 0.161). Of the metabolic hormones, fasting leptin was positively associated with both greater liking (*P* = 0.023) and wanting (*P* = 0.021) responses. Moreover, these relationships remained after adjusted fasting leptin values for percentage body fat (liking, *P* = 0.043; wanting, *P* = 0.041), suggesting they were independent of adiposity.

### 3.4. Physiological Correlates of Food Reward: Exercise-Induced Changes

**Figure 1:**
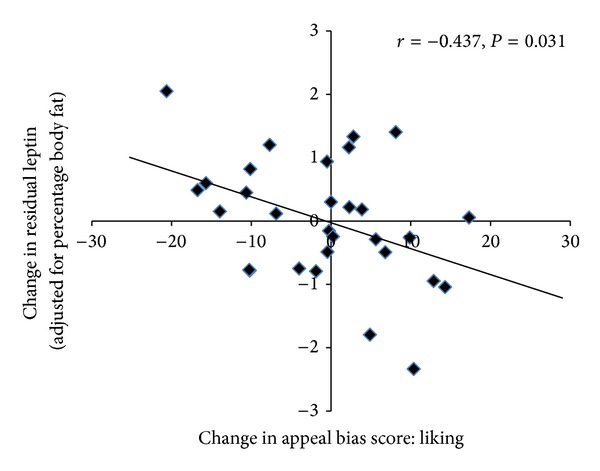
Scatter plot illustrating the relationship between the change in fasting leptin (adjusted for percentage body fat) following the exercise intervention and the change in appeal bias scores for liking for high fat foods (*n* = 32). Positive appeal bias = preference for high fat foods > low fat foods. Negative appeal bias score = preference for low fat foods > high fat foods.

No associations existed between changes in food reward and changes in body composition following the intervention (Table 3). Furthermore, no associations existed between the changes in food reward and the changes in fasting glucose, insulin, HOMA index, or VO_2peak_. However, the change in fasting leptin (absolute or adjusted leptin) was found to be negatively associated with the change in liking for high fat foods following the exercise intervention (*P* = 0.018 and *P* = 0.031, resp.). As can been seen in Figure 1, a decline in fasting leptin following the exercise intervention was associated with increased liking for high fat food. No associations were found between the change in implicit wanting for high fat foods and the change in fasting leptin (absolute or adjusted leptin).

## 4. Discussion

This study examined whether components of body composition and metabolic-related hormones were associated with food reward in overweight and obese individuals during 12 weeks of aerobic exercise. Cross-sectional analyses disclosed associations between body composition (fat mass and fat-free mass), fasting leptin, and food reward. Furthermore, a novel relationship was also disclosed between the change in fasting leptin and the change in explicit liking for high fat foods following the exercise intervention. Specifically, a decline in fasting leptin was associated with an increased liking for high fat foods relative to low fat foods following the intervention. This relationship was independent of changes in fat mass and suggests that leptin may have a key role in mediating changes in food reward during exercise-induced weight loss.

### 4.1. The Effect of Exercise on Body Composition, Metabolism, and Food Reward

The 12-week exercise intervention resulted in significant (but modest) reductions in body mass, fat mass, and percentage body fat, while fat-free mass was preserved at baseline levels. In addition, significant improvements in VO_2peak_ were seen following the exercise intervention. When the changes in food intake and reward were examined, no mean changes in total daily energy intake, explicit liking, or implicit wanting were found. However, it has become clear that examination of the mean (group) response to exercise masked marked heterogeneity in eating behaviour and exercise-induced weight loss following acute [24] and chronic exercise [25–28]. Previous studies have suggested that exercise-induced changes in food reward may mediate compensatory eating behaviour and in turn body weight regulation following exercise [10–12]. However, the physiological correlates of food reward during exercise-induced weight loss have not previously been examined.

### 4.2. Body Composition and Food Reward

Recent evidence has demonstrated the importance of distinguishing explicit perceptions of liking from behavioural operations of wanting, with these components of food reward considered to be separable risk factors in overconsumption and weight gain [5, 6]. During the present study, fat mass and fat-free mass were associated with explicit liking for high fat foods. However, implicit wanting was only associated with fat mass cross-sectionally (i.e., when the baseline, week six, and postintervention measures were combined). These data therefore suggest that fat mass may predict food reward (particularly food wanting) independently of fat-free mass. These findings are consistent with recent observations that fat mass and fat-free mass operated differentially in the control of appetite, with separate roles for fat-free mass in satiation [29] and hunger [19, 30] and fat mass in hedonic eating behaviour traits [31] and neural activation to high energy foods[32]. However, these findings are so far limited to obese individuals and need to be confirmed in a range of different populations, that is, lean versus obese and active versus inactive.

### 4.3. Leptin and Food Reward

It has been suggested that obese individuals display a loss of hedonic control over eating when exposed to highly palatable foods compared to lean individuals [33]. This increase in the susceptibility to overconsumption in obese individuals may be related to increased leptin and insulin resistance (resulting from the excessive accumulation of adipose tissue), which may reduce the sensitivity of short-term appetite control [34, 35]. In the present study, cross-sectional associations were found between explicit liking and implicit wanting for high fat foods relative to low fat foods and fasting leptin, but not fasting glucose, insulin, HOMA, or VO_2peak_. These findings are supported by Raynaud and colleagues [36], who examined the relationships between body composition, serum leptin, insulin, and self-reported palatability of a high CHO breakfast in a sample of predominantly obese adults. A positive relationship was noted between serum leptin and palatability, but not insulin and palatability, and this association remained after controlling for BMI or fat mass. In the present study, the cross-sectional associations indicated that a greater implicit wanting for high fat foods was associated with greater* ad libitum *food intake, suggesting that differences in food reward are expressed behaviourally through differences in food intake (data not reported). However, it should be noted that as the test meal design employed in the present study incorporated both fixed-energy and* ad libitum* meals, the measures of daily energy intake in the present study do not reflect “true”* ad libitum* daily intake.

Interestingly, the present study also disclosed a novel relationship between the changes in fasting leptin and explicit liking for high fat foods following the exercise intervention, with a decline in fasting leptin associated with an increase in liking for high fat foods relative to low fat foods. This relationship is consistent with the proposed role of leptin in food reward, in which leptin is thought to tonically inhibit brain reward pathways [34]. It is hypothesised that a reduction in leptin would act to increase the sensitivity of reward brain circuitry, potentially increasing the motivation to consume highly palatable energy dense foods via its action on dopamine reuptake transporters [37]. Furthermore, leptin's role as an adiposity signal is well established [34], with a decline in leptin thought to stimulate increased hunger and, in turn, food intake, via a downregulation in the hypothalamic expression of anorexigenic neuropeptides, such as proopiomelanocortin and alpha-melanocyte stimulating hormone, and an upregulation in the expression of orexigenic neuropeptides, such as neuropeptide Y and agouti gene-related peptide [38, 39]. Importantly, the present data suggest that a decline in leptin may also promote a greater perceived liking for high fat foods, thereby helping to further promote increased food intake and the restoration of energy homeostasis.

The present findings are in keeping with the idea that leptin is primarily a “starvation” signal rather than a “satiety” signal [40]. While a decline in leptin is thought to promote increases in hunger and food intake [39], an increase in fat mass and leptin does not appear to exert a proportional downregulation in eating [40]. As such, it could be argued that the inhibitory action of fat mass (and associated adiposity signals) on food intake is actually weaker at higher levels of fat mass, and this asymmetry may reflect increased “leptin resistance” with obesity. Indeed, leptin resistance may account for the positive (cross-sectional) correlations between fasting leptin and food reward seen in the present study. However, a decline in leptin (independent of fat mass) during the exercise intervention was also found to be associated with increased explicit liking for high fat foods. While these findings may initially appear contradictory, it has been argued that it is the fall in circulating leptin below a critical (and individualised) threshold level that triggers corrective hypothalamic responses to restore energy homeostasis [41–43]. Theoretically, increased leptin sensitivity resulting from the exercise intervention could have made individuals more sensitive to perturbations in peripheral leptin concentrations, with a decline in leptin perceived by the brain in some individuals as a state of relative leptin deficiency despite an actual surplus of stored energy, that is, fat mass [41–43]. However, clearly this can only be speculated, upon and the precise role of leptin and leptin resistance in food reward remains an important area for future research.

It has previously been reported that the change in leptin (independent of fat mass) during weight loss was negatively associated with the changes in subjective appetite [44]. These observations were made in the context of a 12-week weight loss program in which subjects lost an average of 7 kg fat mass (through diet and exercise). The present intervention on the other hand resulted in a relatively modest 2.2 kg loss of fat mass. Therefore, the subtle effects of the exercise intervention on food reward are perhaps unsurprising. It should be noted that a role for leptin in the hedonic control of food intake during exercise-induced weight loss is a novel hypothesis, and, as such, further work is needed to examine the physiological correlates of food reward in more targeted research. Nevertheless, these findings are consistent with other recent observations that some individuals experience a greater than expected decline in resting metabolic rate following exercise-induced weight loss, and this compensatory downregulation in resting metabolic rate was again associated with a decline in fasting leptin (independent of fat mass). Importantly, those individuals who experienced a compensatory downregulation in resting metabolic rate also experienced a concomitant upregulation in food intake during exercise-induced weight loss [45].

The present study has some limitations that deserve comment. When interpreting the findings of the present study, it is important to note that a nonexercise control condition was not included. However, the observed improvements in body composition, VO_2peak_ are unlikely to have occurred independent of the exercise intervention. Furthermore, due to the need to measure body composition and metabolism at standardised time points during the exercise intervention, no control was made for menstrual cycle phase in female participants. This may have contributed to the variability seen in food reward, as studies have shown that eating behaviour and food reward are influenced by the phases of the menstrual cycle [46, 47].

## 5. Conclusion

Through the concurrent measurement of physiological and behavioural components of energy balance, this study has disclosed novel relationships between food reward, body composition, and metabolic-related hormones in overweight and obese individuals. Cross-sectional relationships were found between measures of explicit liking and both fat mass and fat-free mass. However, only fat mass was found to be associated with implicit wanting, suggesting that aspects of body composition may differentially affect the separate components of food reward. Independent of adiposity, a positive relationship between fasting leptin and liking and wanting for high fat food was demonstrated. Furthermore, a decline in fasting leptin following the exercise intervention was found to be associated with an increase in liking for high fat relative to low fat foods. Taken together, these findings suggest a dynamic role for fasting leptin as a regulatory signal of food reward during exercise-induced weight loss.

## Figures and Tables

**Table 1:** Body composition and metabolic values during the 12-week exercise intervention (*n* = 46).

	Baseline	Week six	Postintervention	Delta Δ	*P* value
Body mass (kg)	88.21 (2.04)	87.39 (2.00)	86.49 (2.04)	−1.72 (0.41)	0.000*
Fat mass (kg)	35.71 (1.34)	34.48 (1.35)	33.49 (1.43)	−2.23 (0.38)	0.000*
Fat mass index (kg/m^2^)	12.61 (0.52)	12.17 (0.53)	11.85 (0.56)	−0.76 (0.14)	0.000*
Body fat (%)	40.33 (1.13)	39.24 (1.16)	38.43 (1.22)	−1.90 (0.32)	0.000*
Fat-free mass (kg)	52.48 (1.43)	52.91 (1.41)	53.00 (1.39)	0.52 (0.17)	0.081
Fat-free mass index (kg/m^2^)	18.25 (0.31)	18.40 (0.30)	18.41 (0.30)	0.17 (0.62)	0.009*
VO_2peak_ (mL·kg^−1^·min^−1^)	33.33 (1.17)	37.45 (1.08)	39.16 (0.09)	5.83 (0.95)	0.000*
Fasting glucose (mmol·L^−1^)	4.93 (0.15)	4.88 (0.17)	4.73 (0.19)	−0.20 (0.25)	0.415
Fasting insulin (ng·L^−1^)	1034.32 (106.24)	918.77 (105.33)	991.34 (113.24)	−42.98 (82.94)	0.230
HOMA index	3.18 (0.31)	2.92 (0.31)	3.02 (0.33)	−0.16 (0.25)	0.554
Fasting leptin (ng·L^−1^)	38318.80 (4832.26)	369923.92 (4612.41)	32102.87 (5333.58)	−6215.93 (3076.37)	0.023*

Delta Δ: baseline to postintervention change. VO_2peak_: maximal aerobic capacity. HOMA: homeostatic model of assessment. *Significant difference between baseline and postintervention (*P* < 0.05).

**Table 2:** Changes in food intake, explicit liking, and implicit wanting for high fat versus low fat foods during the 12-week exercise intervention (*n* = 46).

	Baseline	Week six	Postintervention	Delta Δ	*P* value
Total daily EI (kcal·day^−1^)	2949.29 (79.15)	2877.24 (92.77)	2892.81 (88.11)	−56.48 (60.15)	0.438
Explicit liking (appeal bias score)	−0.20 (2.25)	−1.08 (2.16)	−0.85 (2.02)	−0.65 ( 1.72)	0.919
Implicit wanting (appeal bias score)	1.10 (4.18)	−2.56 (4.47)	−3.17 (3.98)	−4.27 (2.58)	0.114

EI: energy intake; Delta Δ: baseline to postintervention change. Positive appeal bias score = preference for high fat foods > low fat foods. Negative appeal bias score = preference for low foods > high fat foods.

**Table 3:** Pearson partial correlation coefficients (controlling for sex) between food reward and the cross-sectional and exercise-induced changes in body composition and fasting metabolic-related hormones.

Body composition and VO_2peak_			Metabolic hormones		
	Liking	Wanting		Liking	Wanting
BM	0.393**	0.417**	Glucose	0.019	0.060
ΔBM	−0.251	0.116	ΔGlucose	−0.014	0.077
FM	0.341*	0.414**	Adjusted Glucose	−0.267	−0.336
ΔFM	−0.196	0.004	ΔAdjusted Glucose	−0.039	0.061
FMI	0.265	0.351*	Insulin	−0.236	0.311
ΔFMI	−0.223	−0.016	ΔInsulin	−0.206	−0.216
BF%	0.212	0.324*	Adjusted Insulin	0.155	0.194
ΔBF%	−0.210	−0.101	ΔAdjusted Insulin	−0.213	−0.178
FFM	0.295*	0.230	Leptin	0.358*	0.401*
ΔFFM	−0.138	0.265	ΔLeptin	−0.437*	−0.110
FFMI	0.213	0.184	Adjusted Leptin	0.373*	0.370*
ΔFFMI	−0.121	−0.094	ΔAdjusted Leptin	−0.378*	−0.159
VO_2peak_	−0.224	−0.231	HOMA	0.213	0.008
ΔVO_2peak_	−0.178	−0.179	ΔHOMA	−0.123	−0.151
			Adjusted HOMA	0.090	0.139
			ΔAdjusted HOMA	−0.124	−0.152

VO_2peak_: maximal aerobic capacity; FM: fat mass; FMI: fat mass index; FFM: fat-free mass; FFMI: fat-free mass index; %BF: percentage body fat; HOMA: homeostatic model of assessment. Delta Δ: baseline to postintervention change.*P < 0.05; **P < 0.01. Of note: the metabolic-related hormones have been adjusted for percentage fat mass. Cross-sectional models represent the mean scores on each variable collapsed across baseline, week six, and postintervention.
